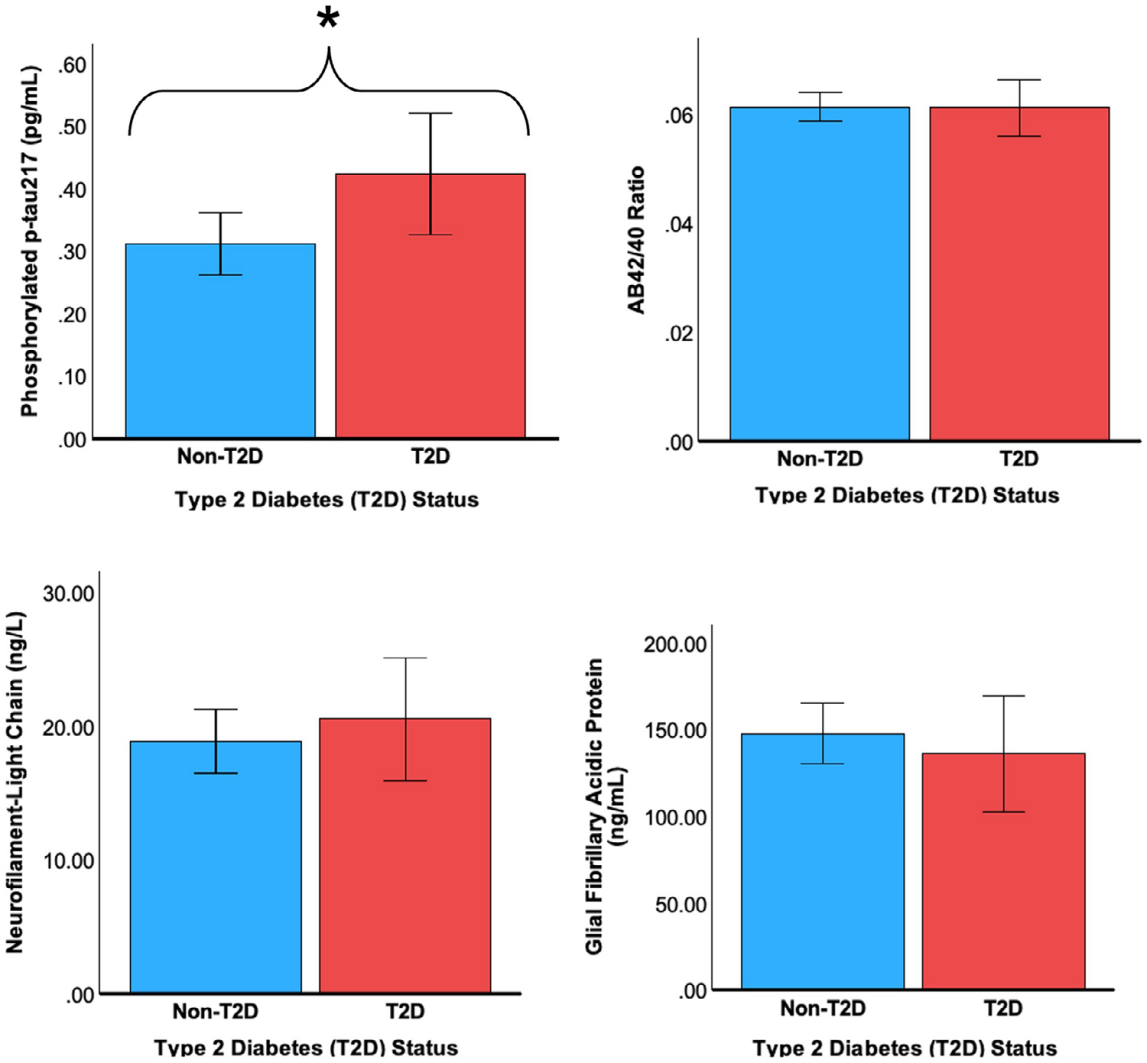# Cognitively Unimpaired Older Adults with Type 2 Diabetes have Higher Levels of Plasma *p* ‐tau217

**DOI:** 10.1002/alz70861_108860

**Published:** 2025-12-23

**Authors:** Darian A. Napoleon, Bernadette A. Fausto, Wiktoria Piaszczynska, Ashley Powell, Stanley Romain, Mc Derby Ceneus, Bisma Iqbal, Andrea Dickantone, Salma Abedullah, Maureen Gichura, Harrison W Chan, Fanny M Elahi, Henrik Zetterberg, Kaj Blennow, Nicholas Ashton, Miray Budak, Steven K. Malin, Patricia Fitzgerald‐Bocarsly, Mark A. Gluck, Robert Perna

**Affiliations:** ^1^ Rutgers University–Newark, Newark, NJ USA; ^2^ Icahn School of Medicine at Mount Sinai, New York, NY USA; ^3^ Hong Kong Center for Neurodegenerative Diseases, Hong Kong, Science Park China; ^4^ Wisconsin Alzheimer's Disease Research Center, University of Wisconsin‐Madison, School of Medicine and Public Health, Madison, WI USA; ^5^ Department of Neurodegenerative Disease, UCL Institute of Neurology, Queen Square, London UK; ^6^ Department of Psychiatry and Neurochemistry, Institute of Neuroscience and Physiology, the Sahlgrenska Academy at the University of Gothenburg, Mölndal, Gothenburg Sweden; ^7^ Clinical Neurochemistry Laboratory, Sahlgrenska University Hospital, Mölndal, Västra Götaland län Sweden; ^8^ UK Dementia Research Institute at UCL, London UK; ^9^ University of Gothenburg, Department of Psychiatry And Neurochemistry, Institute of Neuroscience And Physiology, The Sahlgrenska Academy At The University Of Gothenburg, Mölndal Sweden; ^10^ Department of Psychiatry and Neurochemistry, Institute of Neuroscience and Physiology, The Sahlgrenska Academy, University of Gothenburg, Mölndal Sweden; ^11^ Rutgers, The State University of New Jersey, New Brunswick, NJ USA; ^12^ Rutgers New Jersey Medical School, Newark, NJ USA

## Abstract

**Background:**

Alzheimer's disease (AD) and type 2 diabetes (T2D) share common pathophysiological mechanisms and cognitive sequelae. A potential mechanism by which T2D raises AD risk among aging adults relates to alterations in the medial temporal and prefrontal cortices (Napoleon et al., 2024). However, the relationship of hyperglycemia with neuropathological biomarkers of AD is understudied. Here, we examined the effect of T2D status on blood‐based neuropathology biomarkers in a cohort of cognitively unimpaired older adults of African ancestry.

**Method:**

Cognitively unimpaired older adults (N = 134; 71.87±7.06y; 78.4% female) were recruited from the *Aging & Brain Health Alliance* at Rutgers University–Newark. Individuals were categorized based on T2D status using hemoglobin A1c (≥6.5%, n=28) or non‐T2D (≤6.4%, n=106). Blood samples were analyzed for the following neuropathology biomarkers: plasma *p* ‐tau217 (an early marker of AD neuropathology, elevated in individuals with amyloid‐β [Aβ] deposition); neurofilament‐light chain (NfL, a neuronal cytoplasmic protein that accumulates proportionally to the degree of axonal damage in a variety of neurology disorders; Aβ42/40 ratio (decreased levels are reflective of higher AD risk); and glial fibrillary acidic protein (GFAP; provides structural stability to astrocytes; elevated levels serve as a non‐specific biomarker for neurological diseases). ANCOVAs were used to examine differences between groups on neuropathology markers with age, gender, waist‐to‐hip ratio, and diabetes prescription medication use (0=no, 1=yes) as covariates.

**Result:**

Participants with T2D had significantly higher *p* ‐tau217 levels (η_p_^2^ = 0.03, *p* = .043), independent of covariates. There were no significant group differences on NfL levels (η_p_^2^= 0.01, *p* = .530), Aβ42/40 ratio (η_p_^2^= 0.01, *p* = .96), nor GFAP (η_p_^2^= 0.01, *p* = .54).

**Conclusion:**

T2D was associated with higher levels of plasma *p* ‐tau‐217 compared with those who do not have T2D. This suggests T2D may promote early pathological changes that raise AD risk in cognitively unimpaired older adults (Ashton et al., 2022). These results build on our earlier work demonstrating that T2D and associated hyperglycemia may lower medial temporal lobe cognitive function in cognitively unimpaired older adults, which may be mediated by increased AD neuropathology.